# Proof of concept of an experimental prototype for the prevention of parastomal hernia

**DOI:** 10.1007/s13304-024-01898-0

**Published:** 2024-05-27

**Authors:** María Elena García-Manzanares, Ignacio Zaragoza-García, Mercedes Avilés-Escudero, Beatriz Alonso-Cortés Fradejas

**Affiliations:** 1 Servicio de Cirugía General, Aparato Digestivo y Trasplante de Órganos Abdominales, Hospital Universitario 12 de Octubre, Madrid, Spain; 2 Department of Nursing, Faculty of Nursing, Physiotherapy and Podology, Complutense University of Madrid, Madrid, Spain; 3 Care Research Group (Invecuid), 12 de Octubre Hospital Institute of Health Research (imas12), Madrid, Spain; 4 12 de Octubre Hospital Institute of Health Research (imas12), Madrid, Spain; 5 iPhysio Research Group, Faculty of Health Sciences, Universidad de León, 24401 Ponferrada, León Spain

**Keywords:** Ostomy, Parastomal hernia, Stoma support garments, Prevention

## Abstract

**Purpose:**

The aim of this study was to analyse the complications and problems associated with the use of an experimental prototype designed for the prevention of parastomal hernia (PSH), one of the most frequent complications in ostomates.

**Methods:**

A single-centre, non-comparative, proof-of-concept interventional pilot study of an experimental prototype designed to be used in conjunction with an abdominal compression binder to prevent PSH was conducted. The “Ostomy Fixation Device for Hernia Prevention” (patent P201531826) is a semi-rigid ostomy protector, to be used in conjunction with a compression binder. It is designed to adapt to the dimensions of standard ostomy bags from different brands and serves to transmit, in a localised manner, the support coming from the compression binder in the peristomal area without putting pressure on the collection bag. The main outcome measures were efficacy, safety, and patient-users’ opinion/perception.

**Results:**

Ten patients were studied for 12 months. Mean age was 61 years (± 11.59), 70% (7) were male, 80% (8) ostomised for colorectal cancer, 90% (9) underwent planned surgery and 80% (8) had a colostomy. Efficacy: the incidence of HPE was 10% (1). Safety: no participant experienced pain, discomfort, itching, stinging, leakage, pouch detachment, allergy to components, or injury to the stoma or peristomal skin due to rubbing or pressure. 90% (*n* = 9) were considered “very satisfied” or “satisfied” with the device.

**Conclusions:**

An innovative device designed in collaboration between healthcare professionals and end-users has been shown to be safe and effective in reducing PSH in the group of ostomates studied.

## Introduction

Ostomy surgery can significantly impact the quality of life of affected patients as it causes important physiological, emotional, and relationship changes, in addition to requiring the learning of new self-care habits [1, 2]. Likewise, the creation of a stoma is not a risk-free procedure, and its execution is associated with a high rate of complications [3], amongst which the parastomal hernia (PSH) should be pointed out. Its prevalence is estimated to exceed 30% after 12 months, 40% after 2 years, and 50% or more on a long-term follow-up [4].

The development of PSH is associated with risk factors related to the patient and the surgery [5–7], as well as the lifestyle and the amount of moderate/intense physical exercise carried out [8]. The treatment of PSH represents a significant challenge, which is why its prevention is important. Some authors [8] propose a programme that includes lifestyle advice, abdominal exercises, and the use of abdominal compression binder. The European Hernia Society [4] states that these garments can improve the symptoms of PSH, avoiding the risk of enlargement and/or strangulation of the hernia, although no experimental studies that have shown such effectiveness have been found.

The origin of the prototype described here arose from the need of an ostomy patient who, presenting moderate PSH, sought to protect and contain the peristomal area. Not having found a device on the market to achieve this goal, he decided to create a homemade prototype. With the support of the Ostomy Unit and the Innovation Support Unit of the Research Institute of the 12 de Octubre University Hospital (*i* + 12), the design was improved, resulting in the so-called “Ostomy fixing device for hernia prevention” with Spanish patent P201531826, granted and current.

## Materials and methods

**Fig. 1:**
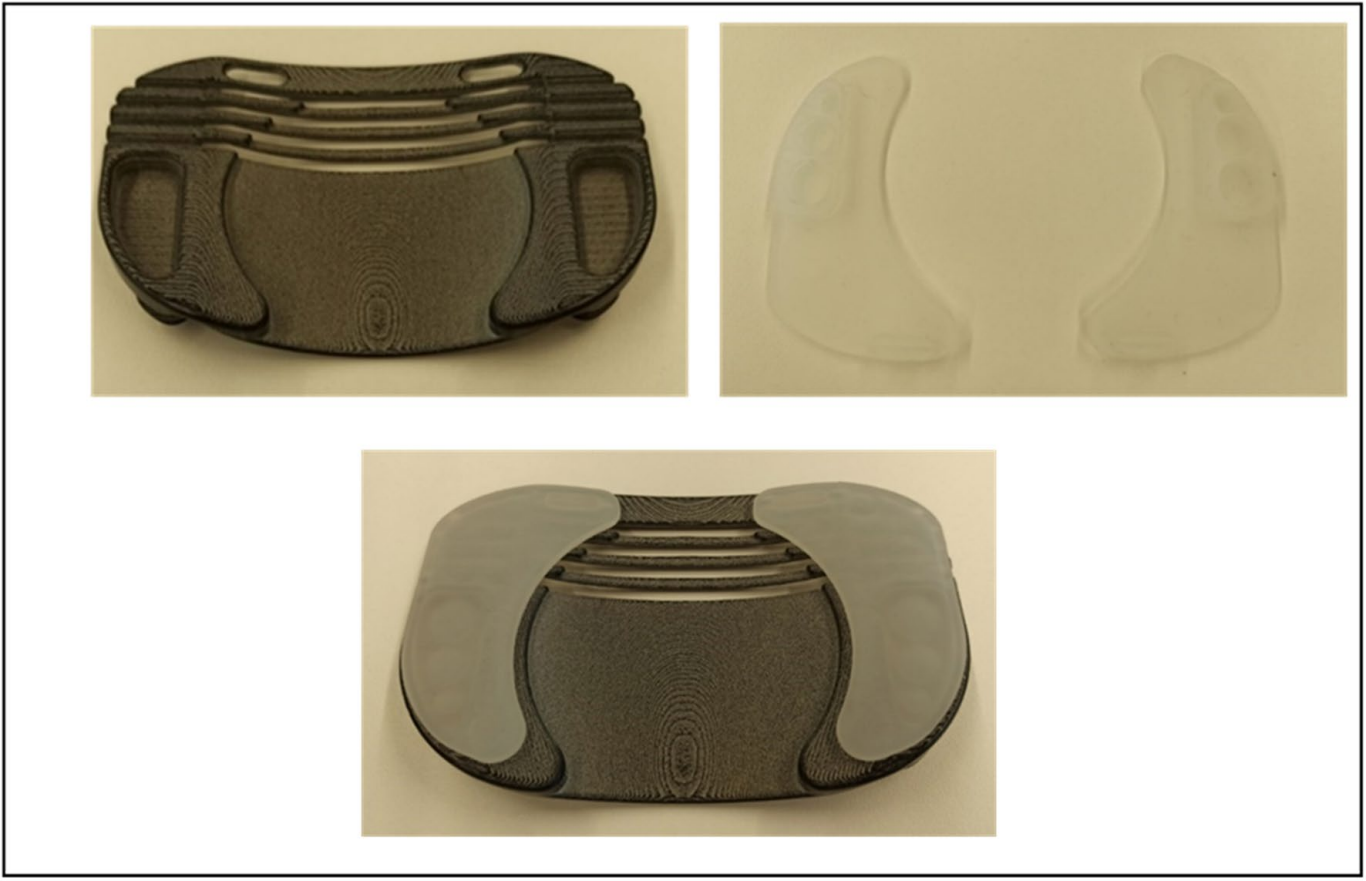
Photographs of the ostomy base with the pads assembled and disassembled

**Fig. 2:**
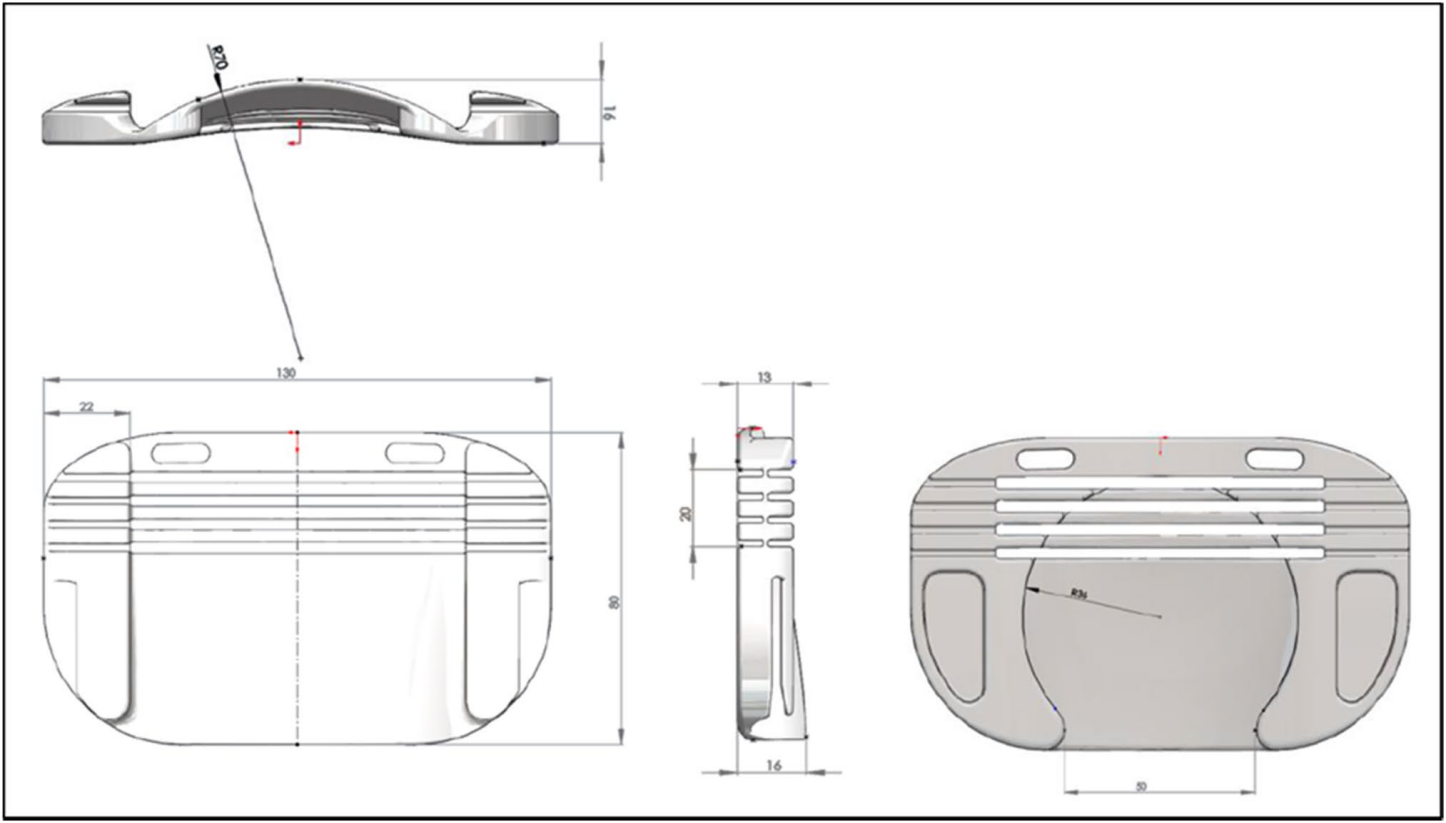
Diagram with the general dimensions of the ostomy base

**Fig. 3:**
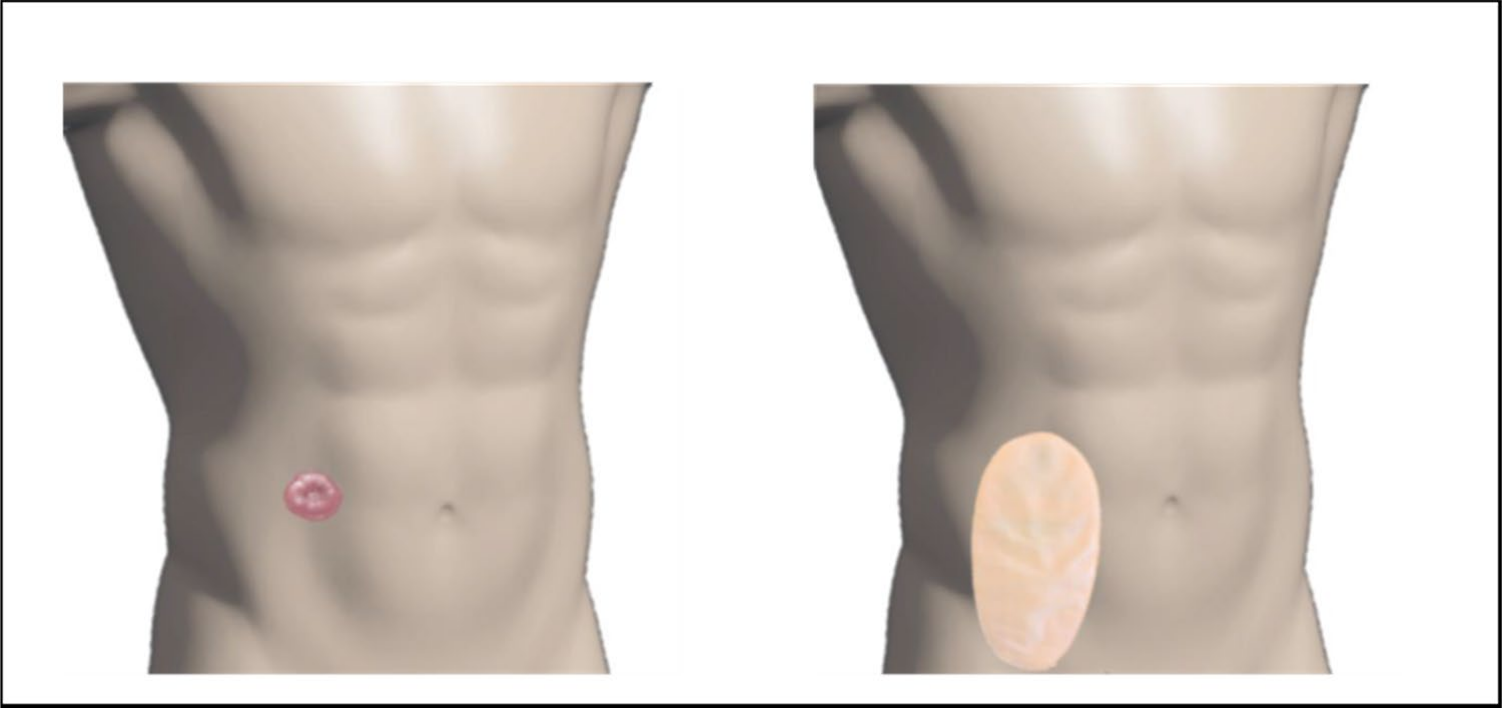
Normal placement of the stool collection bag on the stoma, prior to placing the prototype (ostomy base) and compression garment

**Fig. 4:**
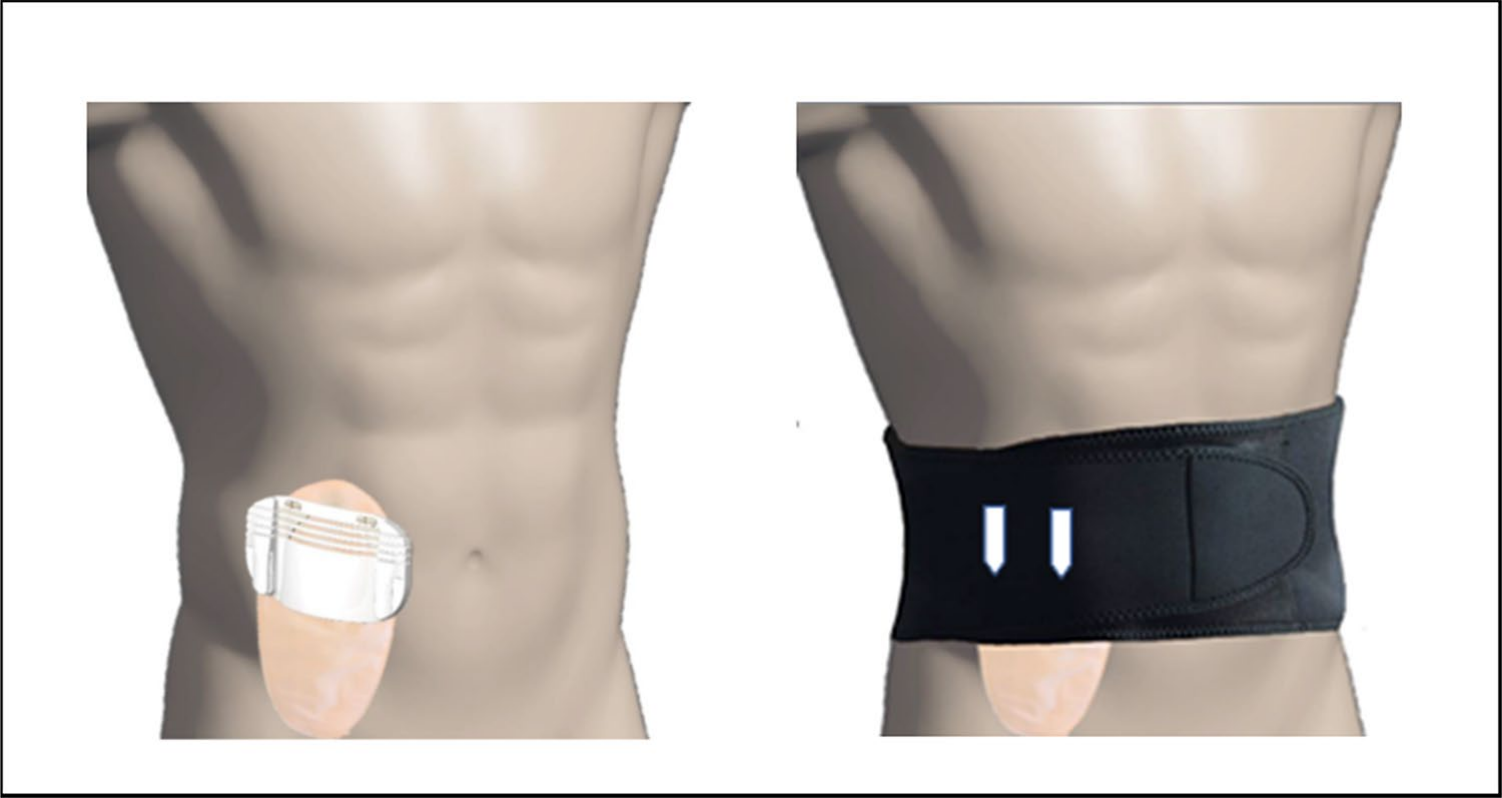
Arrangement and placement of the prototype (ostomy base) on the ostomy bag and the stoma, and its attachment to the abdominal compression garment

The “Ostomy Fixing Device for Hernia Prevention” is a semi-rigid ostomy protector, designed to be worn together with a compression garment. It is designed to adapt to the dimensions of standard ostomy bags from different market brands and serves to locally transmit the support from the compression garment on the peristomal area without putting pressure on the collection bag. The design possesses an ergonomic curvature that avoids pressure on the stoma mucosa or the collection bag. It is manufactured with HP-MultiJet Fusion 3D fast printing prototyping technology and biocompatible PA12 material. Internally, it is shaped like a keyhole to direct the effluent towards the collection bag, and it is covered with identically shaped hypoallergenic silicone pads made of TM6MED medical grade material, which are adjusted using clasps incorporated into the piece itself. Externally, it has openings in the upper area that allow the correct operation of the anti-odour/anti-gas filter of the collection bag. Laterally, it has grooves that provide flexibility to the base (Figs. 1 and 2). The prototype was used together with a securing band/abdominal compression garment already on the market, choosing for each patient a fitting size for their measurements. On the upper external part, the device has notches that allow it to be attached to the compression garment, avoiding the use of Velcro or other easily deteriorated materials (Figs. 3 and 4). The four figures have been adapted from the Technical Document Ostomy Fixing Device for Hernia Prevention (6298/02), by C.T. Tekniker, 2019.

### Study design

A single-centre, non-comparative, proof-of-concept, interventional pilot study of an experimental prototype designed to be worn together with an abdominal compression garment and aiming at the prevention of PSH was conducted. A 12-month follow-up was scheduled for each patient. The enterostomal therapist (ET) evaluated, together with the responsible surgeon, the suitability of each candidate in the study. The information document was delivered during the preoperative consultation with the ET. After accepting participation in the study and signing the informed consent, the ET conducted an initial interview and physical examination. After surgery, the ET performed an in-hospital visit and gave the patient the prototype, the compression garment, and instructions for use. At the 2-, 6-, and 12-months follow-up visits, the ET again conducted an interview and physical examination, and, in the last one, the patient’s assessment of the prototype was also collected through an ad-hoc questionnaire (Table 1).

**Table 1:** Prototype assessment questionnaire

Opinion on the design	Very bad	Bad	Adequate	Good	Very good
How do you rate the shape of the prototype?					
How do you rate the size of the prototype?					
How do you rate the colour of the prototype?					
How do you rate the material of the prototype?					
How do you rate the flexibility of the prototype?					
How do you rate the internal skin contact areas?					
How do you rate the ventilation area of the prototype?					
How do you rate the space between the bag and the base?					
How do you rate the adjustment of the prototype to the compression belt?					
			Yes	No	Sometimes
Does it allow the anti-gas/anti-odour filter to function correctly?					
Does it allow intestinal contents to be evacuated into the ostomy bag?					
Does it limit the capacity of the ostomy bag so that it requires more frequent changes than usual?					
Does it limit the capacity of the ostomy bag in a way that requires the use of a larger size ostomy bag than you normally use?					
Do you think a leakage of the intestinal contents is more likely to happen using the device on top of the ostomy bag?					
**Comfort**	Very bad	Bad	Adequate	Good	Very good
How do you rate the comfort of the prototype?					
1. Standing					
2. Sitting					
3. Walking					
4. Doing light physical activities					
5. Doing moderate/intense physical activities					
6. Lying down					

Adherence to treatment					
How many days did you use the prototype?					
How many hours per day did you wear it?					
**Safety**					
Why did you remove it?					
1. Pain					
2. Discomfort					
3. Itching					
4. Stinging					
5. Leakage					
6. Bag detachment					
7. End of use					
8. Other causes (explain)					
How was the peristomal skin when you removed it?					
1. Normal					
2. Reddened					
3. Irritated					
4. Eroded					
5. Reddened, but regained its normal colour in less than 1 h					
**General assessment**	Very bad	Bad	Adequate	Good	Very good
What is your overall level of satisfaction with the prototype (both pieces)?					
How do you rate the use of the prototype (both pieces) under clothing (discretion)?					
How do you like the two pieces (prototype + compression garment) to be two independent elements?					

### Patients

Were included in the study patients over 18 years of age that had a new stoma with a foreseeable duration of more than 12 months and that were under follow-up in the hospital’s Ostomy Unit. In addition, they had to be able to use the device by themselves, understand the study procedures, and complete the questionnaires. Patients with peristomal mesh, postoperative reoperation due to complications, and/or those with abdominal and/or inguinal hernia prior to the ostomy were excluded. Opportunity sampling was carried out, including patients who agreed to participate voluntarily and did not have exclusion criteria.

### Endpoints

#### Efficacy

The main efficacy outcome consisted of the presence or not of a PSH. Its diagnosis was established by physical examination of the peristomal area, both in standing and supine position, at rest and during manoeuvres to increase abdominal pressure [9]. The information from the Computed Axial Tomography (CAT scan) was added, using the proposal in the European Hernia Guide for the classification of PSH [4].

#### Opinion on the design

The questionnaire contained several questions to assess the design in general and specific terms that were evaluated using a 5-point Likert-type scale, from “very bad” to “very good.” For other questions, the options “yes”, “no” and “sometimes” were given.

#### Comfort

The questionnaire included questions assessing the comfort of the device in three positions (standing, sitting, and walking) and during physical activities (light or moderate/intense). They were evaluated using a 5-point Likert-type scale, from “very bad” to “very good.”

#### Adherence to treatment

The questionnaire included two questions for the patient to assess this aspect: “How many days did you use the prototype?” and “How many hours a day did you wear the prototype?”.

#### Safety

Safety was assessed based on the absence of three potential risks of the prototype. The first was allergy to the components, which was considered unlikely as all materials were medical grade and biocompatible. Secondly, the presence of pressure injuries on the peristomal skin was assessed and, finally, pressure or friction injuries on the stoma mucosa were evaluated. The device was considered safe given that it does not exert direct pressure on the peristomal skin, as it always rests on the ostomy bag, and that the pressure exerted is that generated by the compression belt. It was postulated that the pressure exerted by the prototype would be less than that exerted using a convex appliance indicated in cases of retracted stomata. These devices exert significant pressure on the peristomal area and approximately 30% of ostomates use them [10]. In the same way as these appliances require a periodic review/reassessment of their suitability and relevance by an expert professional, we proposed a close monitoring of our patients to determine the incidence of this possible complication. Answers to two questions included in the patient’s device assessment questionnaire were also considered (“Why did you remove the device?” and “How was the peristomal skin when you removed the prototype?”). The integrity of the peristomal skin was assessed using the validated SACS scale (from the Italian Studio sulle Alterazioni Cutanee Peristomali) [11].

#### Overall satisfaction

This part was evaluated through questions on 4 aspects linked to the satisfaction with the prototype and the abdominal compression garment when used together. They were measured using 5-point Likert scales, from “very satisfied” to “very dissatisfied” for the assessment of general satisfaction, and from “very good” to “very bad” for the assessment of the other two aspects.

### Statistical analysis

A descriptive analysis of the variables was carried out, detailing the quantitative ones through their measures of central tendency, mean or median, and their measures of dispersion, standard deviation or range, respectively. The primary variables of interest, encompassing efficacy, opinion of device use, comfort, adherence to treatment, safety, and satisfaction with the innovative device, were subjected to quantitative analysis, with frequencies and percentages employed to illustrate the results. The relationship between variables was studied by applying Student’s t test and Chi-square test, as appropriate. The REDCap® application was used with a database designed by the IT Service of the Health Research Institute of the hospital under study. The data were analysed with the SPSS® version 26 program.

### Ethical considerations

The study was approved by the Hospital Ethics Committee (CEIm No.: 19/028). The principles established by the Declaration of Helsinki were taken into consideration. All patients gave written consent to participate in the study and the data were treated anonymously.

## Results

### Baseline characteristics

Ten patients, who used the prototype for 12 months, were studied. The mean age was 61 years old (± 11.59) and 70% (*n* = 7) were men.

Regarding the clinical data, 80% (*n* = 8) had had an ostomy for colorectal cancer, 90% (*n* = 9) had had a scheduled surgery and 80% (*n* = 8) had had a colostomy. Analysing the risk factors for PSH, 70% (*n* = 7) had some risk comorbidity: (50% (*n* = 5) Arterial Hypertension, 30% (*n* = 3) Diabetes, 20% (*n* = 2) Inflammatory Bowel Disease, and 10% (*n* = 1) Chronic Obstructive Pulmonary Disease). 50% (*n* = 5) had undergone abdominal surgeries prior to the ostomy. 80% (*n* = 8) presented a higher risk because they had undergone surgery for colorectal cancer and because they had had a colostomy (Table 2).

**Table 2:** Clinical data of the studied sample

Variable	
Previous abdominal surgeries, *n* (%)	
Yes	5 (50%)
Surgery diagnosis, *n* (%)	
Oncological	8 (80%)
Inflammatory bowel disease	2 (20%)
Comorbidities, *n* (%)	
Diabetes	3 (30%)
Respiratory disease	1 (10%)
Inflammatory bowel disease	2 (20%)
AHT	5 (50%)
Others	2 (20%)
Surgery scheduling, *n* (%)	
Scheduled	9 (90%)
Stoma marking, *n* (%)	
Yes	9 (90%)
Surgical procedure, *n* (%)	
Abdominoperineal amputation	5 (50%)
Total/subtotal colectomy	3 (30%)
Ileostomy	2 (20%)
Stoma type, *n* (%)	
Colostomy	8 (80%)
Ileostomy	2 (20%)
Peristomal surgical wound infection, *n* (%)	
No	9 (90%)

### Efficacy

We found an PSH incidence of 10% (*n* = 1) (Table 3). It should be noted that no patient had peristomal mesh placed to prevent PSH during surgery. The only patient with PSH was Case 9 and the diagnosis was established after 12 months by physical examination and CAT scan. He had no symptoms except for the peristomal bulge. After being evaluated by his surgeon, a conservative management of the PSH was considered: monitoring, reducing body weight, and continuing to use the compression garment and the prototype. The participant stated that he felt a greater sensation of support with the prototype than by just wearing the compression garment.

**Table 3:** Presence of hernias (PSH and others) throughout the follow-ups in the sample studied

	Admission	2 months	6 months	12 months
Case 1	No	No	No	No
Case 2	No	No	No	No
Case 3	No	No (midline hernia diagnosed by CAT scan)	No (midline hernia diagnosed by CAT scan)	No (midline hernia diagnosed by CAT scan)
Case 4	No	No	No (hernia in the insertion area of a previous stoma diagnosed by CAT scan + physical examination)	No (hernia in the insertion area of a previous stoma diagnosed by CAT scan + physical examination)
Case 5	No	No	No (bulge)	No (bulge)
Case 6	No	No	No	No
Case 7	No	No	No (midline hernia diagnosed by CAT scan + physical examination)	No (midline hernia diagnosed by CAT scan + physical examination)
Case 8	No	No	No (bulge)	No (bulge)
Case 9	No	No	No	Yes. Type I (diagnosed by CAT scan + physical examination)
case 10	No	No	No	No

In two cases, the presence of a midline incisional hernia was detected by the CAT scan: Case 3 (after 2 months) and Case 7 (after 6 months). In Case 4, an incisional hernia was detected, after 6 months and via CAT scan and physical examination, where there had been a previous stoma. Cases 5 and 8 presented a bulge in the peristomal area, although the diagnosis of PSH could not be confirmed during follow-up.

### Opinion on the design

**Fig. 5:**
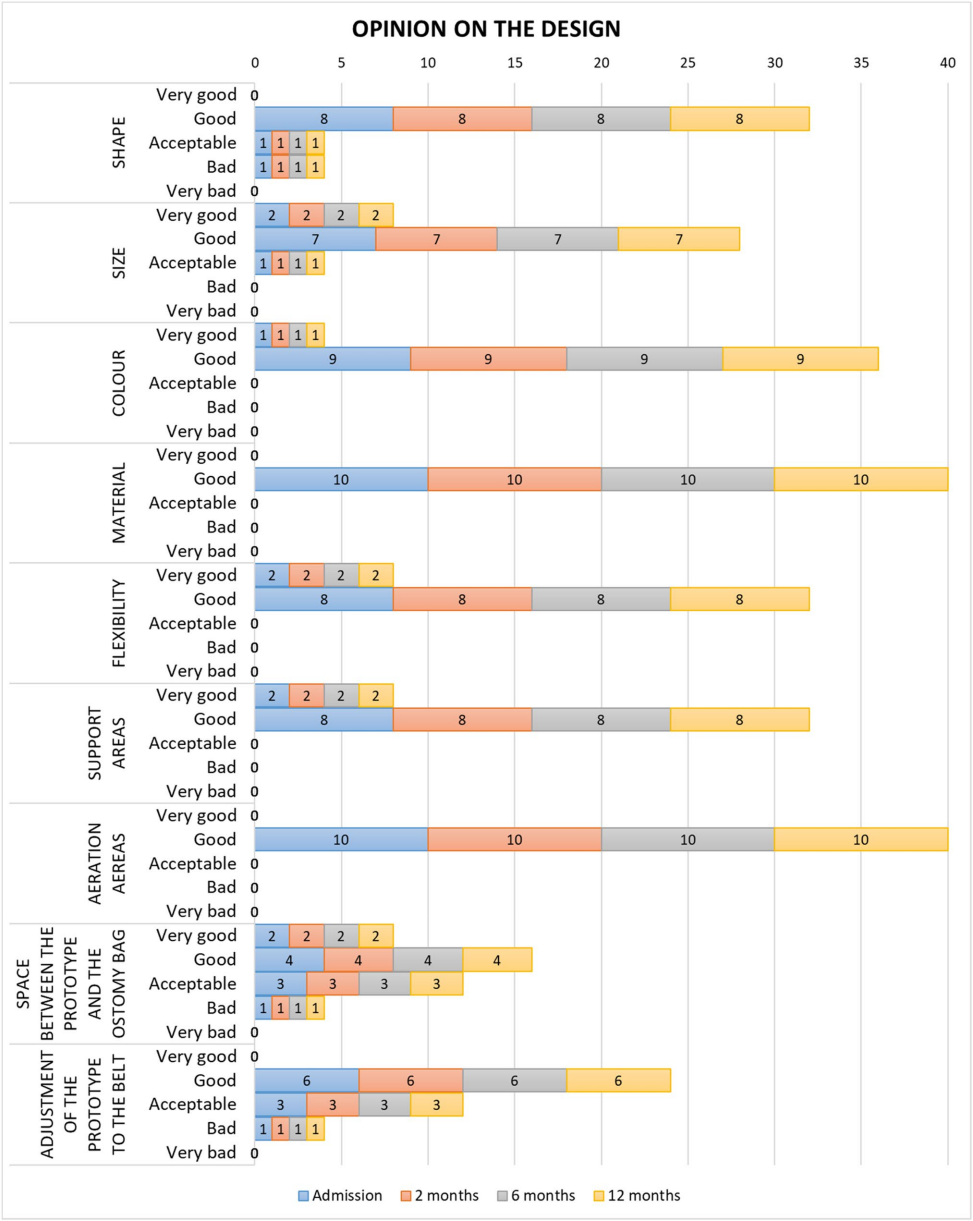
Assessment of the characteristics of the prototype by the sample studied by visits

Between 80% (*n* = 8) and 100% (*n* = 10) of users considered the prototype as “very good” or “good” in relation to shape, size, colour, material, flexibility, support areas, aeration area, space between the prototype and the ostomy bag, and regarding the adjustment of the prototype to the belt (Fig. 5). Only one participant (Case 1) rated the form as “bad”. The patient had a very protruding stoma, but he still used the prototype throughout the entire follow-up without it causing problems to the stoma mucosa or the peristomal area.

### Comfort

**Fig. 6:**
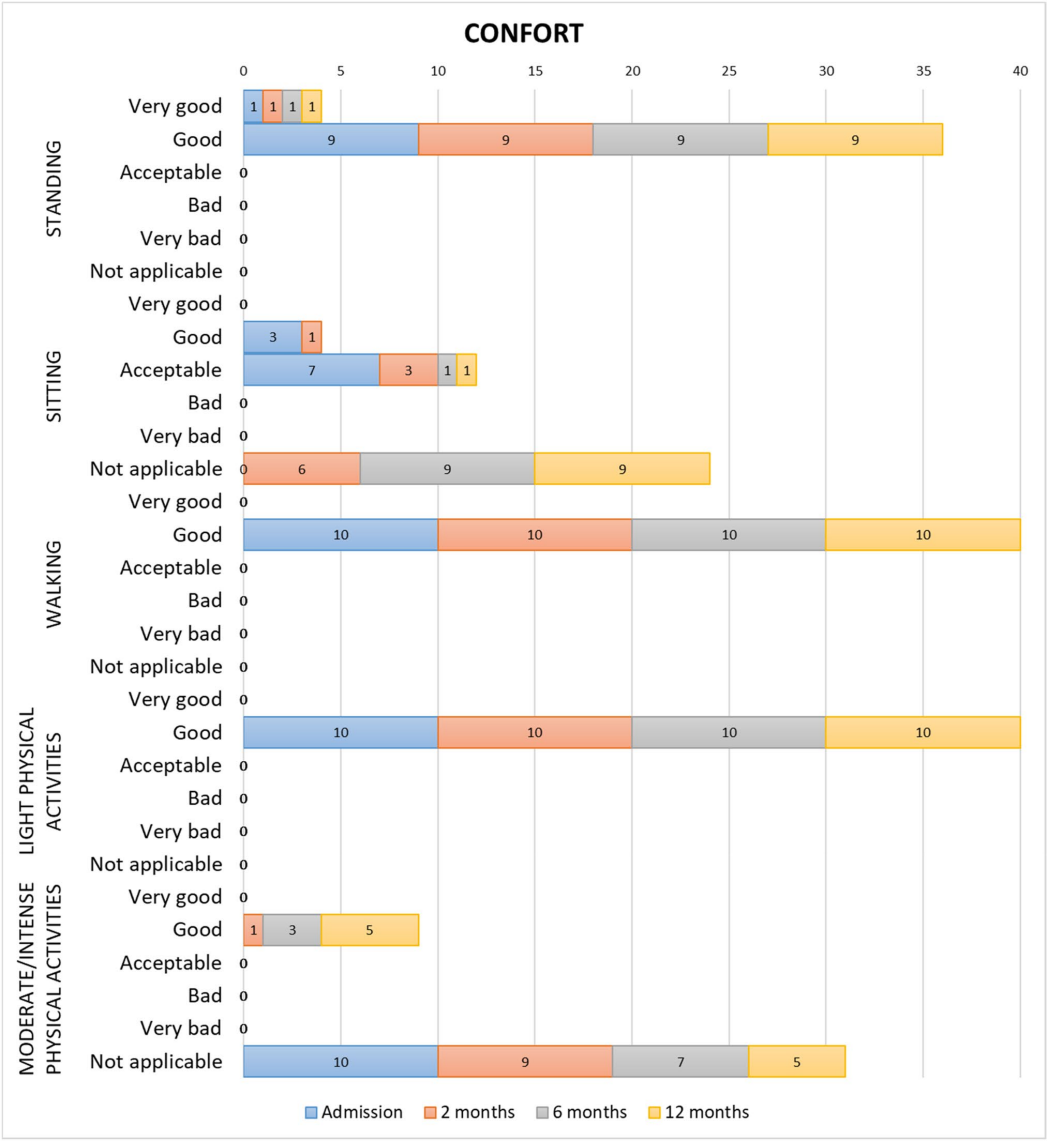
Assessment of the prototype in the sample studied by visits and in different positions and activities/activity level

Ninety per cent (*n* = 9) of the patients considered the use of the prototype whilst standing as “very good” or “good”, reaching percentages of 100% (*n* = 10) whilst walking and doing light physical activities. All participants who did moderate/intense physical activities rated it as “good” whilst doing it (Fig. 6).

### Adherence to treatment

The adherence to the use of both the prototype and the compression garment was 100% (*n* = 10) of the participants. They commented that they used it every day, with an average use of 8 h (mean 7.6 ± 2.12) (Table 4).

**Table 4:** Time of use of the prototype during follow-up in the sample studied

	Admission	2 months	6 months	12 months	Daily average	SD	Mean
CASE 1	4	8	6	8	6,5	± 1,91	7
CASE 2	8	8	8	8	8	± 0,00	8
CASE 3	8	6	6	8	7	± 1,15	7
CASE 4	8	4	4	4	5	± 2,00	4
CASE 5	8	6	4	6	6	± 1,63	6
CASE 6	6	4	8	8	6,5	± 1,91	7
CASE 7	24	12	6	8	12,5	± 8,06	10
CASE 8	8	8	4	8	7	± 2,00	8
CASE 9	10	10	10	8	9,5	± 1,00	10
CASE 10	8	8	8	8	8	± 0,00	8

### Safety

No patient presented injuries to the peristomal skin or stoma related to the use of the device, an aspect that was verified by the ET during the follow-ups. No patient removed the device due to pain, discomfort, itching, stinging, leakage, or bag detachment. It is important to keep in mind that the prototype was applied on stomas with different degrees of protrusion on the skin, on ileostomies and colostomies, and that one of the patients even used the prototype, with medical consent, on a herniated peristomal area, without suffering any of the aforementioned complications or others.

### Overall satisfaction

**Fig. 7:**
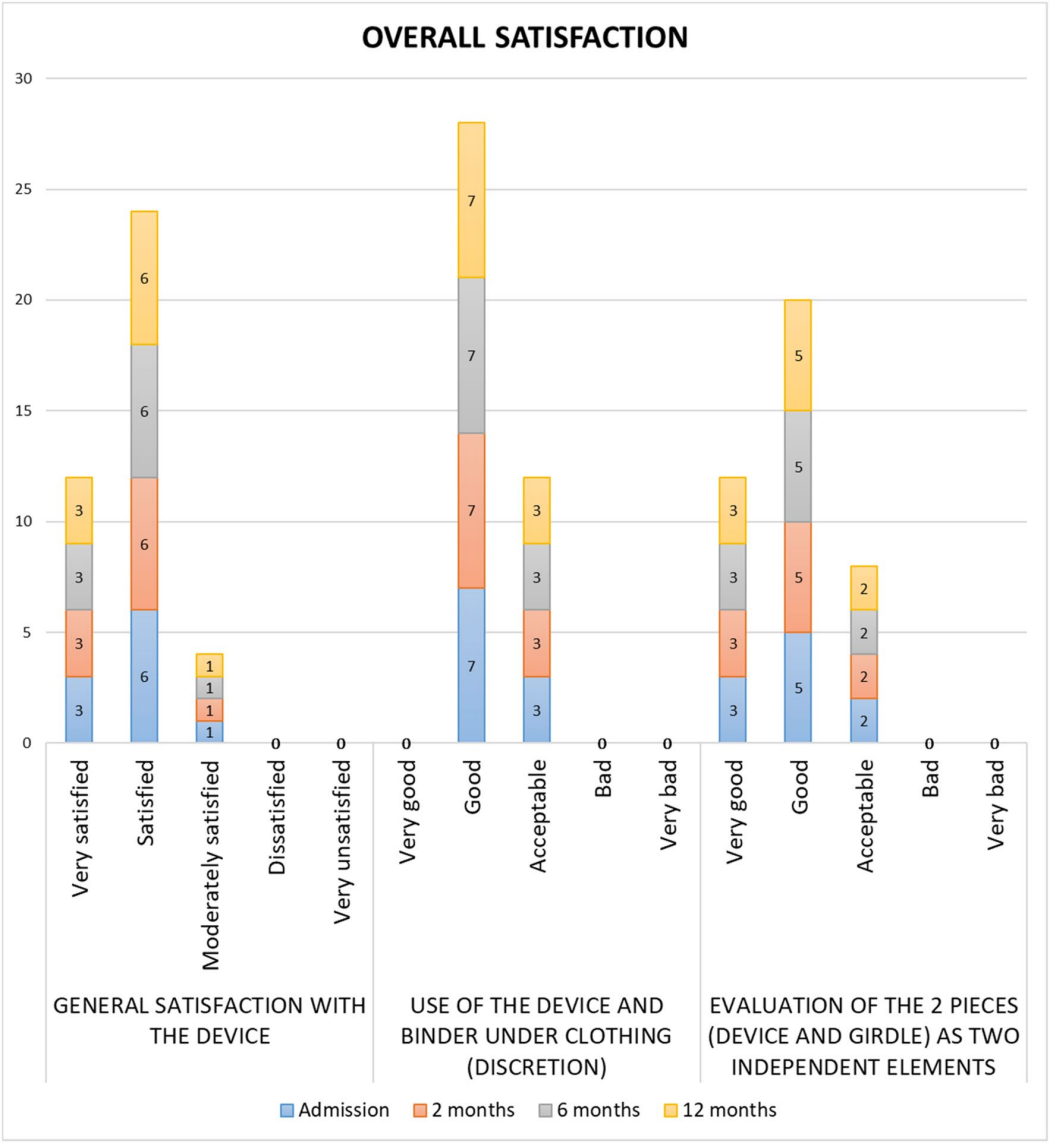
Overall satisfaction with the device in the sample studied by visits

Ninety per cent (*n* = 9) considered themselves “very satisfied” or “satisfied” with the device. Seventy % (*n* = 7) considered the use of the device and the compression garment together “good” in terms of the feeling of discretion and 80% (*n* = 8) considered “very good” or “good” the fact that both pieces (prototype and compression garment) were two independent elements (Fig. 7).

## Discussion

This pilot study was designed to measure the efficacy, safety, adherence, and satisfaction of an innovative device designed for the prevention of PSH based on the idea of an ostomy patient. A sample of ten ostomates was obtained over a 12-month follow-up.

The incidence of PSH in our study (10%) was lower than that of 30% established in the first year after performing the ostomy [12]. Besides, it must be considered that, in our study, physical examination and CAT scan were combined for diagnosis, which is considered the Gold Standard test for the PSH diagnosis. In studies in which it is diagnosed in the same way, the incidence of PSH can even be higher than 86% [13, 14]. To highlight the preciseness of our study, it is also important to mention that the way of performing the physical examination and classifying PSH was previously established according to a validated system, something that is not determined in many of the published studies on the incidence of PSH [15–18].

In our study, the incidence of incisional hernia (including PSH, considered as such) was of 40%, coinciding with that described by other authors [19, 20]. It is noteworthy that the cases of participants who suffered non-parastomal incisional hernia (cases 3, 4, and 7) did not simultaneously present PSH, which makes us think of a possible protective effect of the experimental prototype. Furthermore, it must be considered that, in our study, no patient had peristomal mesh placed to prevent PSH during surgery.

We believe that our good safety results in the use of the device, based on the total absence of injuries to the peristomal skin or stoma, may have been influenced by the design and material of the prototype, but also by the close monitoring by the ET, as well as the correct self-care procedures learned by the patients. It is also important to highlight that an individualised adjustment of the compression garment was made for each patient. Despite the absence of problems in our research, we believe it is appropriate to highlight the fact that, in the first study published on the experience of using support garments in ostomy patients [21], participants linked their use to effluent leaks and odours. This caused concern and social inhibition. Effluent leaks are one of the biggest problems for ostomates and usually cause injuries to the peristomal skin [22–24], reason why we believe it is important to point out that the design of our prototype allows the placement of the compression garment without putting pressure on the collection bag and, therefore, avoiding said problems. In favour of our study, it should also be noted that all participants used the same type of abdominal compression garment, which was not the case in the study carried out by Hubbard et al. [21], in which where included indiscriminately users of *abdominal compression binder* described as “support belts” and “support underwear” (vests, pants, girdles, and trousers). This makes it extremely difficult to generalise their results as it evaluates the effects of compression garments with very different characteristics.

The excellent opinion in relation to the comfort of the device evaluated in all positions of use is an advance on the data presented by Hubbard et al. [21], whose participants considered compression garments uncomfortable due to the sensation of pressure and the mobility restrictions, as well as the pain caused on the stoma, reasons for which they were used for a short period of time.

Regarding treatment adherence, an aspect of great interest for professionals, it is worth noting how, in this study, patients were informed and advised in a personalised way on the use of the prototype and the most appropriate compression garment, which was handed over taking into account weight, height, abdominal perimeter and location of the stoma, as recommended by The Association of Stoma Care Nurses UK [8]. This way of acting and the good adherence achieved is in line with the findings of Borglit et al. [25], who showed that success in using this type of clothing depended on whether the person had received professional guidance and individualised information. Furthermore, the users’ feeling of helping the prevention of a major complication such as PSH may also have favoured our good adherence, as was reported in the study by Hubbard et al. [21].

Regarding the opinion on the design, Hubbard et al. [21] also highlighted the importance of the patient participating in certain decisions about the colour of the garment, fabric, width, or way of placement, as was the case in our study. The aspects of the prototype that the participants considered could be improved were the space left between the prototype and the ostomy bag, as well as the attachment of the prototype to the compression garment (girdle). This adjustment, which was made by taking out the flaps of the prototype through an incision made in the fastening area between the bands of the compression garment, is, without a doubt, an aspect that could be improved, since this cut became larger with use and the prototype could move easily, requiring the user to move it back into place or make new, smaller incisions.

Regarding the overall satisfaction, we have not found other research with which to compare results, which confirms the novelty of our study and prototype.

## Conclusions

Efficacy, design opinion, comfort, treatment adherence and safety of the innovative device have shown good results in this pilot study. Specifically, the safety of this innovative device has been confirmed, as well as its efficacy in preventing PSH, with only a 10% incidence in 12 months. With the device having been used for a significant average of time (8 h a day), very satisfactory opinions were also obtained regarding its use in different daily living positions, as well as its various manufacturing aspects. Although it did not generate any complications, the patients commented that the space between the prototype and the ostomy bag, as well as the fit of the prototype to the compression garment, were aspects of the device that could be improved. We think that the present report can pave the way for further studies with larger sample sizes as well as longer-term follow-up.

## Data Availability

The datasets generated and/or analysed during the current study available from the corresponding author on reasonable request.
